# Oral Microbiota and the Risk of Gastrointestinal Cancers—A Narrative Literature Review

**DOI:** 10.3390/pathogens13090819

**Published:** 2024-09-23

**Authors:** Kinga Knop-Chodyła, Anna Kochanowska-Mazurek, Zuzanna Piasecka, Aneta Głaz, Ewelina Weronika Wesołek-Bielaska, Kinga Syty, Alicja Forma, Jacek Baj

**Affiliations:** 1 University Clinical Hospital Number 4 in Lublin, Jaczewskiego 8, 20-954 Lublin, Poland; kinga.knop03@gmail.com (K.K.-C.); ewelina_wesolek96@wp.pl (E.W.W.-B.); 2 Stefan Cardinal Wyszynski Province Specialist Hospital, al. Kraśnicka 100, 20-718 Lublin, Poland; aniako19@gmail.com; 3 Saint Queen Jadwiga’s Regional Clinical Hospital Number 2 in Rzeszow, Lwowska 60, 35-301 Rzeszów, Poland; zuz.pia@wp.pl; 4 Faculty of Medicine, Medical University of Lublin, al. Racławickie 1, 20-059 Lublin, Poland; glaz.aneta11@gmail.com; 5 Institute of Health Sciences, John Paul the II Catholic University of Lublin, Konstantynów 1G, 20-708 Lublin, Poland; kinga.syty@kul.pl; 6 Department of Forensic Medicine, Medical University of Lublin, Jaczewskiego 8b, 20-090 Lublin, Poland; 7 Department of Correct, Clinical and Imaging Anatomy, Medical University of Lublin, Jaczewskiego 4, 20-090 Lublin, Poland; jacek.baj@umlub.pl

**Keywords:** oral microbiota, gastrointestinal cancer, oral cancer, gastric cancer, esophageal cancer, colorectal cancer, pancreatic cancer, hepatocellular carcinoma

## Abstract

The human body is colonized by trillions of microorganisms in a symbiotic relationship. The oral cavity represents one of the most abundant microbial habitats in our body. Advances in sequencing techniques provide a more detailed understanding of the oral microbiota and how imbalances between bacteria, the phenomenon of dysbiosis, can affect not only the development of dental caries or inflammation within the oral cavity but also systemic diseases and cancers in distant locations. This narrative review evaluates the relationship between oral microbiota and its impact on gastrointestinal cancers. Using the keywords “oral microbiota ‘AND’ gastrointestinal cancers”, the PubMed Web of Science and Scopus databases were searched for articles published between 2014 and 2024. Based on the review, the relationship between oral microbiota and oral, esophageal, gastric, colorectal, hepatocellular, and pancreatic cancers was described. Potential oncogenic mechanisms exploited by the microbiota such as the production of pro-inflammatory cytokines, induction of abnormal immune responses, and disruption of cell metabolic pathways were assessed. Further research and a thorough understanding of the impact of the oral microbiota on the development of cancers of the gastrointestinal tract may play a key role in their prevention, diagnosis, and treatment in the future.

## 1. Introduction

Bacteria inhabiting the oral cavity have come to be known as the “oral microbiota” [1]. According to the expanded Human Oral Microbiome Database (eHOMD), it consists of more than 700 microbial species. Only 58% have been officially named, and 16% are unnamed, but both groups are culturable. In contrast, up to 26% have not received a name and are not cultivated. The most common types of bacteria in the oral cavity are considered to be the following: *Firmicutes*, *Bacteroidetes*, *Proteobacteria*, *Actinobacteria*, and *Fusobacteria* [2]. The differences in the microbiome depend on the location within the oral cavity. Bacteria freely migrate and move with saliva so that saliva, the mucosa of the tongue, cheeks, palate, floor of the mouth, surfaces, and dental spaces maintain their specific ecosystems [3]. Individual variability has also been demonstrated, depending on age, gender, lifestyle, or geographic location [4]. With the proliferation of 16S rRNA profiling techniques, the taxonomic composition of the microbiome is being recognized. However, it may be necessary to evaluate all microbial genomes using shotgun metagenomic sequencing (SM) techniques to assess the pathogenic potential of bacteria well [5]. It is particularly important to understand the correlation between the microbiota and states of health and disease. It is well known that disorders leading to altered microbial composition and dysbiosis in the oral cavity predispose people to inflammation, caries, or periodontal disease. They may also contribute to tumors not only in the oral cavity and pharynx but also to distant organs [6].

## 2. Methods

This narrative review aimed to summarize the latest literature regarding the relationship between oral microbiota and gastrointestinal cancers. The current literature was reviewed by searching for publications from the period between 2014 and 2024 using the search phrase “oral microbiota AND gastrointestinal cancers”. The literature review included the following databases: PubMed, Web of Science, and Scopus.

Three authors independently assessed the eligibility and quality of this study and performed data extraction. References to the retrieved studies were searched manually to ensure the comprehensiveness of this review and to ensure that relevant articles were not omitted. The exclusion criteria were as follows: articles available in a language other than Polish or English and other publication types such as editorials, reviews, posters, and letters. Articles that were duplicates and citations devoid of information relevant to this work were rejected. The literature search included both human and animal studies. In the review, 6 references were based on animal studies, and the remaining 60 were related to humans. Initially, citations were evaluated in terms of the title and abstract. Abstracts were searched with keywords such as the following: oral microbiota, oral cancer, esophageal cancer, gastric cancer, colorectal cancer, pancreatic cancer, and hepatocellular carcinoma. The entire text of the articles found was then analyzed. Finally, 66 articles were included in this review, which discusses in detail the relationship between the oral microbiota and specific gastrointestinal cancers.

## 3. Oral Microbiota and Gastrointestinal Cancers

In order to better understand the issue of gastrointestinal cancers, we summarized the incidence, mortality, and 5-year prevalence rates for oral, esophageal, gastric, colorectal, hepatocellular, and pancreatic cancers in Table 1. Taking the top positions compared to other cancers prompts further research into the prevention, diagnosis, and treatment options for these cancers [7].

The underlying mechanism for the associations between the oral microbiota and risk of gastrointestinal cancer is not completely understood. Several mechanisms are summarized in Table 2.

The oral and intestinal microbiome is well separated due to the presence of the orointestinal barrier, physical distance, and chemical obstacles such as stomach acid and bile. However, it has been shown that the impairment of the orointestinal barrier can enable translocation and inter-organ communication. Moreover, low gastric acidity shifted the composition of the gut microbiome toward the oral microbiome, further indicating the translocation of the oral microbiome into the gut in the case of oral–gut barrier dysfunction. The data suggest that oral microbes can overcome physical and chemical barriers under certain circumstances and potentially translocate to further parts of the gastrointestinal tract and contribute to cancer formation. However, a review of the literature did not find a correlation between the distance from the oral cavity and an increased chance of developing cancer [19].

Many of the data from the studies presented in our review point to a pathological role for periodontal bacteria in the development of gastrointestinal cancers and show a bidirectional relationship between oral and intestinal bacteria, meaning that the treatment of one aspect can positively affect the other. This suggests that the relationship between gastrointestinal cancers and the oral microbiome is mutual [20].

An issue worth raising is the impact of periodontal health and oral hygiene on gastrointestinal cancer development. Chronic inflammation is considered one of the factors in the development of cancer, particularly mediated by the pro-inflammatory cytokine interleukin-20. In addition, poor dental health may increase the production of the carcinogen acetaldehyde by ethanol [1]. Furthermore, the persistence of *Helicobacter pylori* in an unsuitable oral environment favors the migration of *Helicobacter pylori* into the stomach, where it is an important carcinogen [21]. Several studies have also shown that poor oral hygiene and unsatisfactory periodontal health were more frequently observed in colorectal cancer (CRC) patients compared to controls. Studies have suggested that improved oral hygiene could potentially reduce bacterial counts, thereby reducing the risk of bacteria entering the intestines and possibly preventing colorectal cancer [22].

Mutual transmission between the oral and intestinal microbes can affect the ecosystem in both areas, thereby regulating the development of various diseases. Studies suggest a possible interaction between *Helicobacter pylori* and the oral microbiome. The aggregation of *Fusobacterium nucleatum* and *Porphyromonas gingivalis* with *Helicobacter pylori* promotes the colonization of the oral cavity and stomach by oral bacteria. The carious bacterium *Streptococcus mutans* forms a symbiotic biofilm with *Helicobacter pylori* and prolongs its survival in adverse oral conditions. *Porphyromonas gingivalis*, a pathogenic agent of periodontitis, is positively associated with *Helicobacter pylori*, suggesting that *Helicobacter pylori* infection may also promote periodontal disease [23].

Currently under debate is the effect of nutritional therapies on modifying the oral microbiota, which could potentially inhibit the progression of carcinogenesis. Studies suggest that the administration of SCFAs (short-chain fatty acids) may provide early intervention and targeted treatment for gastric cancer. SCFAs exert protective effects on the cell cycle, apoptosis, and immune response through the modulation of cellular pathways (Akt/mTOR and MEK/ERK signaling pathways) and transcription factors (downregulation of NF-κB), as well as epigenetic regulation (inhibition of histone deacetylase inhibitor (HDAC) histone deacetylase activity, DNA methylation, histone phosphorylation, and methylation). One study observed that butyric acid produced by *Porphyromonas gingivalis* inhibits the growth and proliferation of *Helicobacter pylori* in the oral cavity. This suggests that the bactericidal properties of this SCFA may make it suitable for use as a preventive therapy against the movement of *Helicobacter pylori* into the stomach and thus against the development of gastric cancer. Prebiotics such as inulin, fructooligosaccharides (FOSs) and galactooligosaccharides (GOSs), probiotics and fermented foods, and polyphenols may also find use in nutritional therapy [21].

### 3.1. Oral Cancer

Oral cancer is the most common cancer in the head and neck, with oral squamous cell carcinoma (OSCC) histologically accounting for 95% of cases [24].

The factors for the development of OSCC are well known. Among them, we can point to both genetic and environmental ones: smoking and chewing tobacco, alcohol consumption, and human papilloma virus (HPV) infection. However, up to 15% of cancerous lesions are not related to them, prompting researchers to look for other potential causes of OSCC development [25].

Periodontitis is one of the conditions associated with a potentially higher risk of developing oral cancer. Epidemiological data show that the incidence of OSCC was 57.1% in patients with periodontal disease, compared to only 28.6% in patients without inflammation [26]. Among the bacteria that promote chronic infection and the activation of the inflammatory response, we can specifically include *Prevotella intermedia*, *Porphyromonas gingivalis*, and *Fusobacterium nucleatum*, the last two of which additionally promote neoplastic transformation using different mechanisms of action [27]. One of the potential activities of *Porphyromonas gingivalis* is the stimulation of neutrophil extracellular traps (NETs), as demonstrated by immunohistochemical analyses. Although NETs are one of the first lines of defense against infection, their excessive activity also promotes cell invasion, proliferation, and thus the promotion of cancer metastasis [28]. The bacterium promotes the activation of neutrophil chemotaxis in the tumor microenvironment (TME) by increasing the secretion of the chemokine CXCL2, which contributes to tumor transformation and progression. As shown by studies in mice, the inhibition of the CXCL2/CXCR2 signaling axis may become one of the potential treatment targets in OSCC patients [29].

Its oncogenic effect is evidenced by its detection in tissues in OSCC patients, which was associated with faster disease progression and worse survival [30]. In contrast, a study by Lan et al. provides quite the opposite conclusion about the effect of *Porphyromonas gingivalis* on the development of OSCC. It proves its anti-tumor effect by inhibiting the cell cycle in the G2/M phase and decreasing the synthesis of mucin-1 (MUC1) and expression of C-X-C motif chemokine 17 (CXCL17), which stimulates the tumor microenvironment [31]. 

The correlation between periodontitis and associated bacterial dysbiosis and OSCC was also presented in the work of Zhao et al. It was noticed that, in particular, the bacterial taxa *Fusobacterium*, *Dialister*, *Peptostreptococcus*, *Filifactor*, *Peptococcus*, *Catonella*, and *Parvimonas* are more abundant in tumor samples from patients with OSCC. Particular diagnostic value is attributed to *Fusobacterium* [32]. 

A decrease in the entire class of *Firmicutes* bacteria and an increase in the number of *Fusobacteria* have also been observed in OSCC. Yang et al. noted that as the tumor lesion advanced, the levels of certain bacteria like *Streptococcus microbiota*, *Haemophilus*, *Porphyromonas*, and *Actinomyces* decreased, while those of *Fusobacterium periodonticum*, *Parvimonas micra*, *Streptococcus constellatus*, *Haemophilus influenza*, and *Filifactor alocis* increased. Moreover, the most severe stages of OSCC are characterized by the most complex microbiota [33]. Such shifts in bacterial diversity, increasing the abundance of *Fuscobacterium* and decreasing that of *Streptoccocus*, were also demonstrated in the study by Shih-Chi Su et al. The effect of *Streptoccocus* inhibits inflammation, and the resulting dysbiosis promotes the invasion of *Fusobacterium nucleatum* into the mucosa and stimulates the promotion of inflammation. In addition, *Fusobacterium nucleatum* protects the transformed cells in OSCC from immune cells and stimulates further oncogenesis through the activation of Toll-like receptors [34].

### 3.2. Esophageal Cancer

One of the most important factors in esophageal squamous cell carcinoma (ESCC) is poor oral hygiene and correlated changes in the microbiota [5]. 

In a study conducted in Taixing, China, during which the saliva microbiomes of patients with esophageal squamous cell carcinoma, patients with dysplasia, and healthy individuals were examined based on sequencing of the 16S rRNA gene of the V3-V4 region, it was found that the overall microbial diversity in cancer patients was significantly lower compared to those with dysplasia and healthy individuals. In ESCC patients, the reduced number of bacterial genera included *Lautropia*, *Bulleidia*, *Catonella*, *Corynebacterium*, *Moryella*, *Peptococcus*, and *Cardiobacterium*, while bacteria from the genera *Prevotella*, *Streptococcus*, and *Porphyromonas* appeared to be overly abundant [35].

Changes in the oral microbiota in saliva samples from patients with esophageal cancer were also noted by Zhao et al. They found an increased abundance of *Firmicutes*, *Negatividcutes*, *Selenmonadales*, *Prevotellaceae*, *Prevotella*, and *Veillonellaceae* in ESCC patients. On the other hand, *Protecobacteria*, *Betaproteobacerita*, *Neisseriales*, *Neisseriaceae*, and *Neisseria* were fewer [36]. 

There is emerging evidence that an increase in the presence of pathogens such as *Porphyromonas gingivalis* and *Fusobacterium nucleatum* may promote ESCC invasion. It was shown that the abundance of *Fusobacterium nucleatum* was significantly increased in ESCC compared to the esophageal mucosa of healthy subjects. In addition, it was proven that ESCC patients with a higher abundance of this bacterium had significantly shorter survival and worse response to neoadjuvant chemotherapy and had a higher risk of tumor recurrence. A high abundance of *Porphyromonas gingivalis* in ESCC was positively correlated with worse survival, aggressive clinical manifestations, and was associated with the presence of lymph node metastases and advanced clinical stage [37].

### 3.3. Gastric Cancer 

A review of the literature also demonstrates a link between the oral microbiota and gastric cancer (GC). 

Jie Hu et al. studied the relationship between the appearance of tongue plaque and the oral microbiota in patients with gastric cancer. They found that the tongue plaque in GC patients was significantly thicker compared to healthy individuals and that the microbiota of the plaque was correlated with its appearance. Patients with thick raid had significantly lower microbiota diversity compared to healthy individuals with thin raids on the tongue. It was shown that the relative abundance of *Proteobacteria* was significantly lower in GC patients than in healthy patients, which was mainly attributed to the lower abundance of *Neisseria* and *Haemophilus*. The bacteria *Fusobacterium* and *Porphyromonas*, which contribute to periodontal disease, were also less abundant in GC patients. These observations could be used to further analyze tongue plaque as a potential diagnostic source for gastric cancer [38]. Yamamura et al. also showed that *Fusobacterium nucleatum* can contribute to the development of both esophageal and gastric cancer [39].

In China, where the incidence of gastric cancer is high, a study of a 16S rRNA gene sequencing analysis of gastric cancer was conducted, which showed that the microbiota of GCs contained fewer *Proteobacteria* and more *Bacteriodetes*, *Firmicutes*, *Fusobacteria*, and *Spirochaetes* compared to non-malignant tissues. The relative abundance of *Helicobacter* and *Helicobacter pylori* in GC was lower compared to uninvolved gastric tissues. Although *Helicobacter pylori* colonization was present in patients with gastric cancer, the composition of the gastric microbiota did not differ according to the presence of infection [40].

In their study, Sun et al. demonstrated that increased colonization with periodontal pathogens, in particular *Treponema denticola*, *Tannerella forsythia*, and *Actinobacillus*, in the oral cavity of gastric cancer patients reduced the diversity of bacteria in the plaque. In addition, it was shown that a lack of regular flossing was identified as a significant predictor of an increased risk of precancerous lesions in gastric cancer, demonstrating the importance of daily oral hygiene [41].

Other studies show that the oral cavity is another reservoir for *Helicobacter pylori* as well. Although the bacterium is found mainly in the stomach and is the most abundant bacterium in the gastric microbiome, accounting for 40 to 90%, numerous studies have found *Helicobacter pylori* in the oral cavity, including saliva, plaque, tongue plaque, and dental pulp. Yuko Ogaya et al. conducted a study in which bacterial DNA was isolated from 41 adult and 21 pediatric saliva samples, and *Helicobacter pylori* was detected by PCR. The presence of this bacterium was observed in as many as 46.3% of the adults and 38.1% of the children tested [42]. Mutita Wongsuwanlert et al. in their study also confirmed *Helicobacter pylori* colonization in the oral cavity. Strains with the cag A and vac A genes are the most pathogenic oncoproteins involved in colonization and damage to the gastric epithelium. According to the results, attention should be paid to the presence of *Helicobacter pylori* and its virulence gene in the oral cavity. The eradication of such strains from the oral cavity can help prevent the transmission and re-colonization of gastric organs [43]. *Helicobacter pylori* infection plays a key role in mucosal inflammation, induces histological changes, and promotes the degradation of gastric epithelial architecture and integrity, which is considered an important etiological factor in gastric cancer. Communities affected by *Helicobacter pylori* infection are significantly more likely to develop gastric cancer than communities without the disease [44].

### 3.4. Colorectal Cancer

Colorectal cancer (CRC) is one of the most prevalent cancers worldwide and a significant cause of mortality. Recent studies are trying to answer the question of whether the oral microbiome plays a role in the carcinogenesis of colorectal cancer and whether it can be a source of its microbiological and metabolic biomarkers. In order to clarify these questions, Zhang et al. conducted a study in which the structure and function of the oral microbiota were evaluated using collected oral swabs from patients with colorectal cancer, patients with colorectal adenocarcinoma, and healthy volunteers. Differences in the composition and diversity of the oral microbiome were observed between the three groups of patients. There was an increase in the number of bacteria of the genus *Fusobacterium*, especially in the group of patients with colorectal adenoma, suggesting that *Fusobacterium* spp. could potentially be involved in the development of CRC [45].

*Fusobacterium nucleatum* is commonly found in dental plaque but has been shown to be able to migrate from its primary site of colonization to other parts of the body and thus influence the occurrence and development of systemic diseases, including cancer. It plays a role in promoting the initiation and progression of CRC through various mechanisms. It has been shown that through the virulence factor FadA adhesin, it can adhere to and attack tumor cells and promote oncogenic and inflammatory responses, thereby leading to the progression of colorectal cancer. A study using the ApcMin/+ mouse model demonstrated that *Fusobacterium nucleatum* can induce DNA damage and cell growth in CRC through the activation of the E-cadherin/β-catenin pathway [46].

*Fusobacterium nucleatum* nucleic acids are also detected in colorectal cancer tissues. Sequencing studies suggest that intracancerous strains of this bacterium may have an oral origin. The promotion of chemotherapy resistance in CRC tissues by *Fusobacterium nucleatum* may be associated with a poorer prognosis for patients and more frequent cancer recurrence [47].

Studies have shown that *Fusobacterium nucleatum* increases the production of reactive oxygen species (ROS) and inflammatory cytokines (e.g., IL-6 and TNF) in colorectal cancer. This bacterium exhibits immunosuppressive effects by inhibiting human T-cell responses and suppressively modulating the tumor microenvironment. In a multicenter study by Mima et al., 511 CRC tissues were collected from Japanese patients. The presence of *Fusobacterium nucleatum* was detected in 44 (8.6%) participants. Moreover, a positive result was significantly associated with high microsatellite instability (MSI) status [48].

Another example of a bacterium potentially involved in colon cancer carcinogenesis is *Parvimonas micra*. Bergsten et al. identified its two phylotypes (A and B). It has been shown that phylotype A, characterized by hemolytic capacity and adhesive properties, capable of colonizing the colonic mucosa, can induce changes in DNA methylation and thus promote tumorigenesis [49].

Russo et al. conducted an observational study comparing saliva samples from patients with colorectal cancer and adenomatous polyps. Its aim was to look for possible changes in the oral microbiota potentially associated with the progression of polyps to invasive cancer. No significant difference was observed between the two groups of patients. However, in the saliva samples examined, there was a significant increase in the number of bacteria from the Spirochaetota cluster, classes Spirochaetia, order Gracilibacteria, and Absconditabacteriales in the group of patients with T3 or T4 features in the TNM classification, i.e., with a higher stage, compared to the group of patients with T0 to T2 features [50]. To assess whether there are differences in the gut microbiota between patients at different stages of CRC, one cohort study showed that *Parvimonas micra*, *Peptostreptococcus stomatis*, *Solobacterium moorei*, *Gemella morbillorum*, and *Fusobacterium nucleatum* had increased abundance in both the early and late stages, while Actinomyces odontolyticus abundance increased only in early stages [51].

Nearing et al. analyzed the oral microbiome for retrospective and prospective colorectal cancer cases. Differences in microbiome diversity were observed only in retrospective cases. There was a positive association between time since diagnosis and alpha diversity (rho = 0.62, *p* = 0.04). We also tried to assess whether specific ASVs (amplicon sequence variants) are associated with the development of CRC. In retrospective colorectal cancer cases, an increase in ASV classified as *Fusobacterium peridonticum* was observed. Additionally, a decrease in the relative abundance of ASV classified as Streptococcus was observed [52]. Changes in the diversity of the oral microbiome in saliva samples were also found in individuals with Lynch syndrome, which is the most common hereditary colorectal cancer syndrome. These findings suggest a possible role for bacteria in cancer development in patients with a genetic predisposition [53]. Flemer et al. evaluated the usefulness of the oral microbiota as a screening tool for identifying people with colorectal cancer. The oral swab microbiota classification model used distinguished colorectal cancer patients from controls. The sensitivity of detection was 53% with a specificity of 96%. In addition, it was observed that the presence and abundance of oral pathogens in both CRC subjects and healthy participants were negatively associated with the abundance of *Lachnospiraceae* such as *Anaerostipes*, *Blautia*, and *Roseburia.* This may suggest that these bacteria play a protective role against colon cancer, possibly by preventing the colonization of the colon with oral bacteria, including CRC-associated oral pathogens such as *Peptostreptococcus*, *Parvimonas*, and *Fusobacterium* [54]. Also, a study by Rezasoltani et al. revealed that the assessment of the oral microbiota may be a suitable screening test for the early detection of this malignancy, with the potential to provide more accurate results than its fecal equivalent. Therefore, the inclusion of information on the oral microbiota has the potential to improve the effectiveness of current diagnostic tests for the detection of colorectal cancer, but further research is needed to enable reliance on oral microbiota testing in clinical practice [55].

### 3.5. Pancreatic Cancer

Pancreatic cancer remains one of the world’s most aggressive cancers, with its incidence increasing [56]. There are two main types of pancreatic cancer: pancreatic ductal adenocarcinoma (PDAC) and pancreatic neuroendocrine tumors (PanNETs). Regardless of the type, the disease lacks early detection markers and effective therapies, with a poor prognosis. Despite years of research, patients with pancreatic cancer are often diagnosed with advanced stages of the disease [57].

New evidence suggests that the oral microbiota plays an important role in human health, including immune response, carcinogen metabolism, and nutrient digestion. Recent epidemiological studies show that poor oral health and antibodies in the blood against selected oral pathogens increase the risk of pancreatic cancer [58].

Microbiologists have confirmed that the spread of oral microbes into the pancreas occurs by translocation or spreading. This phenomenon has been confirmed in both animal and human models. Researchers believe that the dysbiosis of the oral microbiota precedes the development of pancreatic cancer, rather than developing only after cancer has occurred [59]. According to the current literature, *Porphyromonas gingivalis*, *Fusobacterium*, *Neisseria elongata*, and *Streptococcus* mitis are the leading pathogens among the oral bacteria involved in the pathogenesis of pancreatic cancer.

*Porphyromonas gingivalis* is a pathogenic bacterium involved in chronic periodontitis. Fan et al. made a comparison of the composition of the oral microbiota in samples from 361 individuals who developed pancreatic cancer and 371 healthy participants selected for age, sex, race, body mass index, smoking, alcohol consumption, and diabetes. The study indicated that *Porphyromonas gingivalis* carried a 59% higher risk of pancreatic cancer. Researchers also found higher levels of ATTC 53978 antibodies against *P. gingivalis* in the blood of patients with pancreatic cancer than in healthy volunteers, and they were associated with twice the risk of developing pancreatic cancer. This study brings hope that ATTC 53978 may serve as an indicator of potential pancreatic cancer in the future [60].

Another discovery was made by Qin Tan et al. Their study showed that *Porphyromonas gingivalis* from the oral cavity, implanted and enriched in a tumor-bearing pancreas, promotes pancreatic cancer progression in mouse tumor models. Tumors infected with *Porphyromonas gingivalis* recruit tumor-associated neutrophils in the microenvironment and increase the release of neutrophil chemokines and neutrophil elastase, which promotes pancreatic cancer progression. In addition, *Porphyromonas gingivalis* accumulates in the pancreatic tissues of cancer patients to a greater extent than in adjacent healthy tissues [61].

An interesting discovery was made by Ai-Lin Wei et al. They showed that the saliva microbiome was able to distinguish pancreatic cancer patients from healthy individuals. Compared to healthy controls, carrying *Streptococcus* and *Leptotrichina* was associated with a higher risk of pancreatic cancer. *Veillonella* and *Neisseria* bacteria, on the other hand, were considered protective microorganisms that reduced the risk of pancreatic cancer. Among patients with ductal adenocarcinoma of the pancreas, patients reporting bloating had a higher abundance of *Porphyromonas*, *Fusobacterium*, and *Alloprevotella*, while patients with jaundice had a higher abundance of *Prevotella* [62].

A subsequent epidemiological study proved that *Porphyromonas gingivalis* migrated from the oral cavity to the pancreas in mice and could be detected in human pancreatic epithelial neoplasia lesions. The findings of Elijah Saba et al. proved that *Porphyromonas gingivalis* induces pancreatic ductal metaplasia and alters the composition of the intrapancreatic microbiome. In mice with pancreatic lesions, *Porphyromonas gingivalis* accelerated progression from pancreatic epithelial neoplasia to pancreatic adenocarcinoma [63].

### 3.6. Hepatocellular Carcinoma

Hepatocellular carcinoma (HCC) accounts for as many as 75–85% of cases of primary liver cancer. The main risk factor for HCC is cirrhosis of any etiology. Others are chronic infection with hepatitis B virus (HBV) or hepatitis C virus (HCV), as well as the consumption of aflatoxin-contaminated food, heavy alcohol consumption, smoking, type 2 diabetes, and excessive weight [7].

There are now many studies attempting to provide new markers useful in the diagnosis of hepatocellular carcinoma. It has been discovered, among other things, that oral bacteria such as *Porphyromonas gingivalis*, *Epsilon proteobacteria*, *Actinobacteria*, *Clostridia*, and *Fusobacteria* predominate in patients with HCC, while their numbers decrease with the development of liver disease. The mechanism of the hepatic–gut axis promoting the development of HCC was also studied. It was found that lipopolysaccharides from Gram-negative bacteria combine with TLR-4 and TLR-2 to stimulate T lymphocytes and macrophages to produce cytokines and pro-inflammatory mediators, which can consequently cause an increase in the liver levels of NADPH oxidoreductase 4 (NOX-4) and malondialdehyde and a decrease in the glutathione, catalase, and selenium levels. This cascade of actions can lead to the deterioration of liver function and, moreover, to chronic liver inflammation, which may result in hepatocellular carcinoma in the future [64].

**Figure 1:**
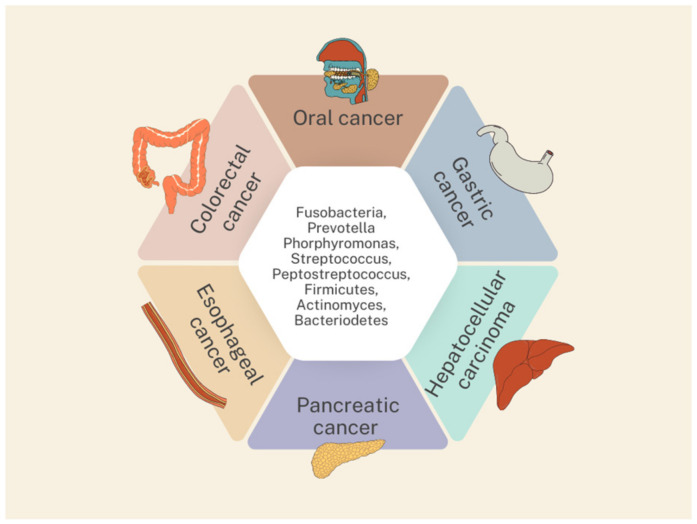
The most important oral microbiota identified contributing to the development of gastrointestinal cancers.

Lu et al. examined the microbiome of the tongue overgrowth of patients with cancer and cirrhosis based on the sequencing of the 16S ribosomal RNA (rRNA) gene. They proved that the diversity of the tongue overgrowth microbiome of patients with liver cancer was significantly higher compared to the microbiome of healthy individuals (*p* < 0.05). The differences were mainly in *Oribacterium* and *Fusobacterium*, which were mainly found in cancer patients [65]. Another study isolated bacteria that predominated in both hepatocellular carcinoma patients and healthy subjects. These included *Firmicutes*, *Proteobacteria*, *Bacteroidetes*, *Actinobacteria*, and *Fusobacteria*. However, there was a significant increase in Firmicutes and Actinobacteria in oral samples from HCC patients compared to controls (*Firmicutes*: 26.81% versus 32.91%; *Actinobacteria*: 9.74% versus 13.38%). On the other hand, the relative abundance of *Proteobacteria* and *Fusobacteria* was significantly increased in control subjects (Proteobacteria: 28.45% versus 21.35%; *Fusobacteria*: 11.11% versus 9.09%) [66]. The composition of the oral bacterial flora has great potential for future use in the diagnosis and prognosis of hepatocellular carcinoma, and the removal of periodontal pathogens may be a new therapeutic option for HCC. To summarize, the contribution of individual bacteria to gastrointestinal cancers is summarized in Table 3. The most important bacteria are also highlighted in Figure 1.

## 4. The Limitations of This Review and Suggestions for Future Studies

The presented manuscript has many limitations. The conclusions are based on preclinical models, cross-sectional study results, and studies on animals. There is also a lack of confirmation of the links between oral dysbiosis and the risk of gastrointestinal cancer in prospective studies. It remains extremely difficult to compile data concerning fluctuations in the oral microbiota depending on age, sex, race, socioeconomic status, oral hygiene, alcohol drinking, smoking, diet, ethnicity, comorbidities, antibiotic use, and genetic factors.

## 5. Conclusions

The relationship between the oral microbiota and the process of carcinogenesis is still under debate. Numerous studies suggest that changes in the oral microbiome can be used as risk markers for the development of gastrointestinal cancers and provide a jumping-off point for their treatment. However, research findings are not consistent, lacking the systematization of aspects related to the composition, abundance, and individual variability in the oral microbiota in health, as well as dysbiosis. Conducting further research, disseminating 16S rRNA and SM techniques, and striving for a further systematization of the microbiota may provide a better understanding of the interrelationships between them and thus allow us to explore the mechanisms underlying systemic diseases and cancers.

## Figures and Tables

**Table 1:** Summary incidence, mortality, and 5-year prevalence rates for oral, esophageal, gastric, colorectal, hepatocellular, and pancreatic cancers [7].

Type of Cancer	Incidence (World Rank)	Mortality (World Rank)	5-Year Prevalence (Europe)
Oral cancer	16th	15th	18.5%
Esophageal cancer	11th	7th	10%
Gastric cancer	5th	5th	11.6%
Colorectal cancer	3rd	2nd	29.4%
Pancreatic cancer	12th	6th	26.7%
Hepatocellular carcinoma	6th	3rd	8.7%

**Table 2:** Mechanisms by which oral microbiota could contribute to gastrointestinal cancer development [1,8,9,10,11,12,13,14,15,16,17,18].

Mechanism	Significant Pathogens	Related Signaling Pathways	
Chronic inflammation	*Porphyromonas gingivalis*	Inducing chronic inflammation and oncometabolite production by activating anti-apoptotic proteins, growth factors, and cytokines that foster cancer growth and dissemination.	[1,8,9,10,11,12]
	*Fusobacterium nucleatum*	Lipopolysaccharides (LPSs) can activate response through Toll-like receptors (TLRs), including TLR2 and TLR4, which can inhibit apoptosis and promote tumor growth.	
Inhibition of immune response	*Fusobacterium nucleatum*	Expansion of myeloid immune cells that inhibit T-cell proliferation and induce T-cell apoptosis.	[1,8,13]
	*Porphyromonas gingivalis*	Protecting tumor cells from NK cells and immune cells through interaction between Fap2 protein and inhibitory immunoreceptor TIGIT (T-cell immunoreceptor with Ig and ITIM domains) on NK and T cells.	
Interference with signaling pathways	*Fusobacterium nucleatum*	Binding to epithelial (E) cadherin via FadA, leading to growth stimulation of human cancer cells.	[8,14,15]
Local metabolism of carcinogens	*Neisseria*	Activating alcohol and smoking-related carcinogens such as acetaldehyde and nitrosamines.	[8,16,17,18]
	*Candida glabrata*		

**Table 3:** A summary of the impact of the oral microbiota on the development of gastrointestinal cancers.

Type of Cancer	The Authors of the Study	Year	Bacteria ↑	Bacteria ↓	
Oral cancer	Zhao et al.	2017	*Fusobacterium*, *Dialister*, *Peptostreptococcus*, *Filifactor*, *Peptococcus*, *Catonella*, *Parvimonas*	No information	[32]
	Yang et al.	2018	*Fusobacterium* *periodonticum*, *Parvimonas micra*, *Streptococcus* *constellatus*, *Haemophilus* *influenza*, *Filifactor alocis*	*Streptococcus*, *Haemophilus*, *Porphyromonas*, *Actinomyces*	[33]
	Su et al.	2021	*Fusobacterium*	*Streptococcus*	[34]
Esophageal cancer	Chen et al.	2015	*Prevotella*, *Streptococcus*, *Porphyromonas*	*Lautropia*, *Bulleidia*, *Catonella*, *Corynebacterium*, *Moryella*, *Peptococcus*, *Cardiobacterium*	[35]
	Zhao et al.	2020	*Firmicutes*, *Negatividcutes*, *Selenmonadales*, *Prevotellaceae*	*Protecobacteria*, *Betaproteobacteria*, *Neisseriales*, *Neisseriaceae*, *Neisseria*	[36]
	Gao et al.	2023	*Porphyromonas* *gingivalis*, *Fusobacterium* *nucleatum*	No information	[37]
Gastric cancer	Hu et al.	2015	*Prevotella*, *Streptococcus*, *Veillonella*, *Actinomyces*, *Leptotrichia*	*Neisseria*, *Haemophilus*, *Porphyromonas*, *Fusobacterium*	[38]
	Yu et al.	2017	*Bacteriodetes*, *Firmicutes*, *Fusobacteria*, *Spirochaetes*	*Proteobacteria*	[40]
	Sun et al.	2017	*Treponema* *denticola*, *Tannerella forsythia*, *Actinobacillus*	No information	[41]
Colorectal cancer	Flemer et al.	2018	*Actinomyces*, *Rothia*, *Veillonella*, *Parvimonas micra*, *Peptostreptococcus* *stomatis*, *Dialister* *pneumosintes*, *Neisseria*, *Prevotella*, *Fusobacterium* *nucleatum*	No information	[54]
	Zhang et al.	2020	*Fusobacterium*, *Prevotella*, *Veillonellla*, *Treponema*, *Phorphyromonas*	No information	[45]
	Rezasoltani et al.	2022	*Eubacterium*, *Bifidobacterium*, *Fusobacterium*	No information	[55]
	Nearing et al.	2023	*Fusobacterium* *peridonticum*	*Streptococcus*	[52]
Pancreatic cancer	Fan et al.	2018	*Porphyromonas* *gingivalis*	No information	[58]
	Wei et al.	2020	*Streptococcus*, *Leptotrichina*	No information	[62]
	Tan et al.	2022	*Porphyromonas* *gingivalis*	No information	[61]
Hepatocellular carcinoma	Lu et al.	2016	*Oribacterium*, *Fusobacterium*	No information	[65]
	Yang et al.	2023	*Firmicutes*, *Actinobacteria*	No information	[66]

## Data Availability

No data were used for the research described in this article.
